# Preparation of Hydrophobic Purple Sweet Potato-Based Intelligent Packaging Films by Stearic Acid Coating and Heat Pressing Treatments

**DOI:** 10.3390/foods14071276

**Published:** 2025-04-05

**Authors:** Xuanzhuo Liu, Fengfeng Xu, Xiaoqian Huang, Jian Sun, Juan Kan, Jun Liu

**Affiliations:** 1 College of Food Science and Engineering, Yangzhou University, Yangzhou 225127, China; 222403113@stu.yzu.edu.cn (X.L.); dx120240257@stu.yzu.edu.cn (F.X.); mz120211966@stu.yzu.edu.cn (X.H.); junliu@yzu.edu.cn (J.L.); 2 Xuzhou Institute of Agricultural Sciences in Jiangsu Xuhuai Area, Xuzhou 221131, China; xzsunjian@jaas.ac.cn

**Keywords:** anthocyanins, color-changeable films, hydrophobic surface, stearic acid coating, heat pressing

## Abstract

The high hydrophilicity of biopolymer–anthocyanin intelligent packaging films seriously limits their applications in high-humidity environments. Here, a surface hydrophobization technique was adopted to overcome the hydrophilicity of purple sweet potato (PSP)-based intelligent packaging films through stearic acid (SA) coating combined with heat pressing treatments. The structural characteristics, physical properties, and color changeability of the films were investigated. After SA coating treatment, the surface of the films was loosely covered by thick SA layers. As compared with the untreated PSP films, the SA-coated films displayed lower transparency, mechanical property, moisture content, surface wettability, anthocyanin leaching potential, and color changeability. When the SA-coated films were further heat-pressed, the SA-coated layers were closely bound to the films. The heat-pressed films had a higher transparency, mechanical property, and water vapor blocking ability than the SA-coated films. Notably, the color and color changeability of the heat-pressed films were affected by the heat pressing temperature. The films heat-pressed at 100 °C showed a vivid purple color and elevated color changeability, whereas the films heat-pressed at 150 °C showed a brown color and lost color changeability. This study demonstrates that SA coating combined with heat pressing is effective in constructing surface-hydrophobized intelligent packaging films.

## 1. Introduction

Food packaging serves as an effective means of maintaining food quality and protecting food from external mechanical damage, environmental stress, and microbial contamination [1]. With consumers’ awareness of environmental protection in mind, biopolymer-based packaging originating from natural polysaccharides and proteins have been considered ideal alternatives to traditional petroleum-based packaging [2]. Nowadays, intelligent packaging techniques are emerging on the foundation of biopolymer-based packaging [3]. In particular, the fabrication of intelligent packaging films by loading plant anthocyanins into biopolymer-based hydrocolloids has become a research hotspot [4]. The prominent function of biopolymer–anthocyanin intelligent packaging films is the real-time monitoring of the quality of food within the packaging, where anthocyanins show color changes if food is spoiled [5]. Since the vast majority of natural biopolymers and plant anthocyanins are hydrophilic substances, biopolymer–anthocyanin intelligent packaging films often undergo anthocyanin leaching in high-humidity environments [6].

To overcome the high hydrophilicity of biopolymer–anthocyanin intelligent packaging films, researchers have attempted to directly incorporate hydrophobic substances such as castor oil [7], curcuma oil [8], and sunflower wax [9] into these films. However, these hydrophobic substances have very limited compatibility with hydrophilic biopolymers and anthocyanins. As a result, the hydrophobicity of intelligent packaging films is only slightly improved by directly added hydrophobic substances [7,8,9]. In recent years, surface hydrophobization has been recognized as an effective approach to enhancing the hydrophobicity of biopolymer-based packaging films [10]. Researchers have successfully constructed biopolymer-based packaging films with hydrophobic surfaces by coating low-surface-energy materials, such as fatty acids, waxes, and clays [11]. Nonetheless, the surface hydrophobization of biopolymer–anthocyanin intelligent packaging films with low-surface-energy materials has rarely been reported [12,13,14].

Stearic acid (SA), a common fatty acid with low surface energy, possesses several advantages (e.g., low cost, non-toxicity, and biocompatibility) in constructing hydrophobic surfaces for biopolymer-based packaging films [15,16]. Recently, some researchers have managed to increase the surface hydrophobicity of biopolymer–anthocyanin intelligent packaging films through SA coating [13,14]. However, the SA-coated layers normally have a weak interfacial binding force with intelligent packaging films, thereby affecting the durability of the films [13,14]. Moreover, the SA-coated layers are unevenly distributed on the surface of intelligent packaging films, which adversely affects the color changeability of intelligent packaging films [14]. Therefore, it is essential to elevate the interfacial binding force and uniformity of the SA-coated layers on biopolymer–anthocyanin intelligent packaging films.

In our previous study, we found that thermally treated purple sweet potato (PSP) tubers were rich in biopolymers (e.g., starch, dietary fibers, and proteins) and highly acylated anthocyanins, which were suitable for producing intelligent packaging films [17,18]. In the present study, we innovatively fabricated PSP-based intelligent packaging films with hydrophobic surfaces through SA coating and heat pressing treatments. First, intelligent packaging films were produced based on the thermally treated PSP powder by casting. Then, PSP-based intelligent packaging films were surface-hydrophobized via SA coating combined with heat pressing treatments, making the SA-coated layers closely bind and uniformly distribute on the surface of the films. The impacts of the SA coating concentration (1% and 5%), heat pressing temperature (100 and 150 °C), and heat pressing time (1 and 2 min) on the structural characteristics, physical properties, color changeability, and shrimp freshness monitoring ability of the films were investigated. This study provides a new approach to constructing biopolymer–anthocyanin intelligent packaging films with hydrophobic surfaces.

## 2. Materials and Methods

### 2.1. Materials and Chemical Reagents

PSP (*Ipomoea batatas* (L.) Lam) tubers were freshly harvested from Xuzhou Institute of Agricultural Sciences (Xuzhou, China). Chemical reagents including sodium alginate, glycerol, SA, ethanol, and 2,2-diphenyl-1-picrylhydrazyl (DPPH) were of analytical-grade purity and were bought from Macklin (Shanghai, China). Fresh shrimps were purchased from Yonghui Superstore (Yangzhou, China).

### 2.2. Preparation of the Thermally Treated PSP

PSP tubers were thermally treated based on the method of Yun et al. [18], with modifications. First, dust was removed from the surface of PSP tubers by tap-water washing. The washed PSP tubers were placed in the LS-35HD autoclave (Xinling Instrument, Zhengzhou, China) and heated at 121 °C for 30 min. The thermally treated PSP tubers were cut into 2 cm sized cubes, lyophilized, and milled into powder. The obtained PSP powder was sieved through a 100-mesh sieve and stored in a refrigerator (–20 °C) for further use.

### 2.3. Preparation of Surface-Hydrophobized PSP-Based Intelligent Packaging Films

First, a PSP-based intelligent packaging film was prepared by casting [17]. PSP powder (1.2 g) was homogenized in 40 mL of distilled water at 6000 rpm for 2 min, which was followed by heating in an LC-OB-5L water bath (Lichen, Shanghai, China) at 95 °C for 20 min. Sodium alginate (0.24 g) and glycerol (0.36 g) were sequentially added to the PSP powder homogenate and mixed thoroughly at 30 °C for 1 h to yield a film-forming solution. The film-forming solution was first degassed in a KQ-200KDE ultrasonic cleaner (Kunshan Ultrasonic Instrument, Suzhou, China) at 100 W for 15 min and was then cast in a leveled mold (10 cm × 10 cm) at 30 °C for 60 h. The obtained PSP-based intelligent packaging film was named the P film.

**Figure 1:**
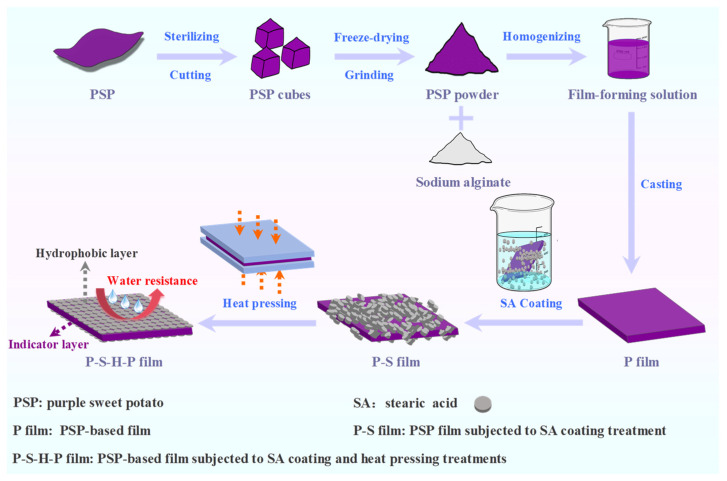
Flow chart for the preparation of PSP-based intelligent packaging films with hydrophobic surfaces via SA coating combined with heat pressing treatments.

Afterwards, surface-hydrophobized PSP-based intelligent packaging films were fabricated by SA coating and heat pressing treatments. Briefly, 1% and 5% SA coating solutions were individually prepared by dissolving 5 g and 25 g SA in 500 mL ethanol at 70 °C for 1 h. The previously prepared P film was soaked in 1% and 5% SA coating solutions for 5 min. The films obtained by 1% and 5% SA coating were named the P-S1 and P-S5 films, respectively. The P-S1 and P-S5 films were then subjected to heat pressing treatment using a DQYB-XMTF-6 heat transfer press machine (Yizhao Machine, Jinhua, China) under different operation conditions, i.e., 100 °C for 1 min, 100 °C for 2 min, 150 °C for 1 min, and 150 °C for 2 min. The resultant films were named the P-S1-P100-H1, P-S1-P100-H2, P-S5-P100-H1, P-S5-P100-H2, P-S1-P150-H1, P-S1-P150-H2, P-S5-P150-H1, and P-S5-P150-H2 films, where P100/P150 represents the heat pressing temperature (100 and 150 °C) and H1/H2 represents the heat pressing time (1 and 2 min). All films were equilibrated at 50% relative humidity (RH) and 20 °C for 48 h. The preparation processes of the surface-hydrophobized PSP-based intelligent packaging films are shown in Figure 1.

### 2.4. Structural Characterization of the Films

#### 2.4.1. Scanning Electron Microscopical Observation

The surfaces and cross-sections of the films were observed using a GeminiSEM 300 scanning electron microscope (Carl Zeiss, Oberkochen, Germany) at 2 kV with 100× and 600× magnifications, respectively. For surface observation, the films were directly fixed on an observation stage through conductive adhesive. For cross-section observation, the films were first fractured in liquid nitrogen and then fixed on an observation stage through a conductive adhesive. The fixed films were sputtered with gold to improve the imaging quality.

#### 2.4.2. Infrared Spectroscopic Analysis

The chemical components within the films were analyzed by a Varian 670 infrared spectrometer (Agilent, CA, USA). A total of 32 cumulative scans were carried out for each film at the resolution of 2 cm^−1^ and the wavenumber range of 4000–400 cm^−1^.

#### 2.4.3. X-Ray Diffraction Analysis

Films were fixed on a test bench with double-sided adhesive and analyzed by a D8 Advance X-ray diffractometer (Bruker, Karlsruhe, Germany). First, a Ni filter was used to achieve a pure Cu-Kα characteristic radiation. Subsequently, films were scanned under the diffractometer with the scan increment of 0.2° and the diffraction range of 5–45°.

### 2.5. Physical Property Measurements of the Films

#### 2.5.1. Color

The photograph of the films was documented using a Redmi K70 smartphone (Xiaomi, Beijing, China). The color of the films was quantitatively analyzed with an SR-62 colorimeter (3nh Technology, Shenzhen, China) by recording the chromatic coordinates (L*, a*, and b*) and color difference (Δ*E*) of the films [19]. The measurements were performed three times for each film.

#### 2.5.2. Transparency

The transparency of the films was tested using a Lambda 35 ultraviolet-visible (UV–Vis) spectrophotometer (PerkinElmer, MA, USA) by scanning film strips (9 mm in width) from 200 to 800 nm [20]. The measurements were performed three times for each film.

#### 2.5.3. Thermal Property

The thermal property of the films was measured using an HTG-1 thermogravimetric apparatus (Henven, Beijing, China) by loading 2.5 mg samples in aluminum crucibles and heating the samples from 50 to 650 °C with a heating rate of 10 °C/min [21]. The measurements were performed three times for each film.

#### 2.5.4. Mechanical Property

The mechanical property of the films was detected by loading film strips (6 cm × 1 cm) onto an STX200 texture analyzer (Yishite, Xiamen, China) and stretching the film strips at a speed of 3 mm/s [22]. The tensile strength (TS) and elongation at break (EAB) of the films were directly read from the texture analyzer (Yishite, Xiamen, China). The measurements were performed six times for each film.

#### 2.5.5. Moisture Content (MC)

The MC of the films was determined by weighing square film samples (with sides 2 cm in length) before and after drying at 105 °C for 6 h [23]. The measurements were performed three times for each film.

#### 2.5.6. Water Vapor Blocking Performance

The water vapor blocking performance of the films was determined by measuring the water vapor permeability (WVP) via a gravimetric method [19]. Square film samples (with sides 4 cm in length) were sealed on permeability cups filled with 10 mL of water (100% of RH). The cups were then placed in an air-tight container (25 °C) loaded with 200 g silica gel desiccant. The film-sealed cups were continuously weighed at 24 h intervals, lasting for 7 days. The measurements were performed three times for each film.

#### 2.5.7. Surface Wettability

The surface wettability of the films was determined by recording the water contact angle (WCA) of water droplets (2 μL) on film surfaces through a GP-50 charge-coupled device camera (Gaopin, Suzhou, China) [24]. The WCA of the films was captured at 20 s intervals, lasting for 2 min. The measurements were performed six times for each film.

#### 2.5.8. Stability in Water

The stability of the films in water was evaluated by placing square film samples (with sides 1 cm in length) in 3 mL of distilled water. The swelling behavior of the film samples was recorded at 2 min intervals, lasting for 10 min. Film leachates were collected periodically and measured for their UV–Vis spectra and DPPH radical scavenging activity [14]. For UV–Vis spectral measurement, 1 mL of film leachate was scanned from 200 to 600 nm using a Lambda 35 UV–Vis spectrophotometer (PerkinElmer, Waltham, MA, USA). For DPPH radical scavenging activity measurement, 1 mL of film leachate was allowed to react with 3 mL of DPPH ethanol solution for 1 h, and then the absorbance of the reaction solution was detected at 517 nm. The measurements were performed three times for each film.

#### 2.5.9. Biodegradability

The biodegradability of the films was determined by burying square film samples (with sides 2.5 cm in length) in natural soil with a burial depth of 8 cm. The temperature and RH of the soil were controlled at 20 °C and 70%, respectively. The morphological changes in film samples were recorded at 4-day intervals, lasting for 24 days [15].

### 2.6. Measurement of the Color Changeability of the Films

The color changeability of the films was evaluated by placing square film samples (with sides of 1.5 cm in length) in pH 3–12 buffers for 60 s and in 0.1 mol/L volatile ammonia for 90 min [25]. The color of the film samples under different acid–base conditions was recorded by a Redmi K70 smartphone (Xiaomi, Beijing, China).

### 2.7. Evalution of the Shrimp Freshness Monitoring Ability of the Films

First, square film samples (with sides of 2 cm in length) were fixed onto the inner lid of a transparent crisper. After loading 180 g fresh shrimp, the crisper was sealed and placed in a refrigerator (4 °C). Throughout the entire period, it was ensured that the film samples were not in contact with shrimps. The color of film samples and the total volatile basic nitrogen (TVB-N) of shrimps were recorded at 24 h intervals, lasting for 6 days. The correlation between the Δ*E* value increase in film samples and the TVB-N of shrimps was analyzed [26].

### 2.8. Statistical Analysis

Data were analyzed by one-way analysis of variance (ANOVA) and Duncan multiple-range tests using SPSS 13.0 (SPSS Inc., Chicago, IL, USA), and results were expressed as mean ± standard deviation (SD) with statistical significance at the level of *p* < 0.05.

## 3. Results and Discussion

### 3.1. Microstructures of the Films

**Figure 2:**
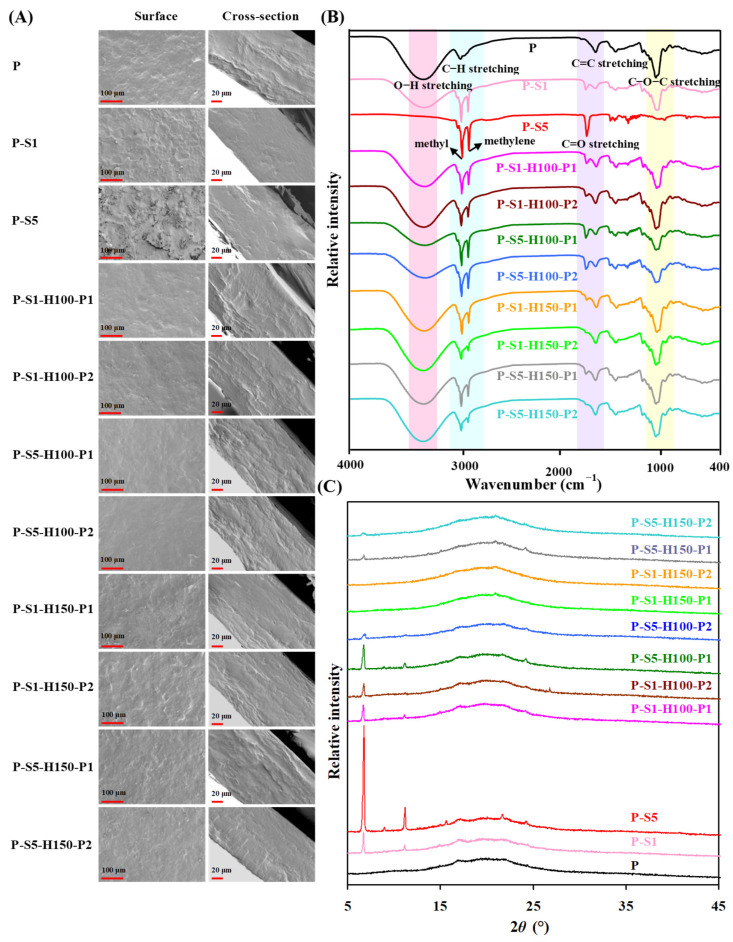
The surface and cross-sectional microstructures (**A**), infrared spectra (**B**), and XRD patterns (**C**) of the films.

The superficial and cross-sectional microstructures of the films are depicted in Figure 2A. The P film prepared from PSP powder showed a rough surface, which was attributed to the insoluble biopolymers (e.g., cellulose and semi-cellulose) in PSP [18]. After the P film was soaked in 1% and 5% SA coating solutions, the surfaces of the obtained P-S1 and P-S5 films were loosely covered by massive SA layers. The massive SA layers were formed by self-assembly [27,28]. As a result, the P-S1 and P-S5 films displayed rougher surfaces than the untreated P film. Since the P-S5 film was produced by soaking in a higher concentration (5%) of the SA coating solution, the P-S5 film was covered by thicker SA layers. After the P-S1 and P-S5 films were subjected to heat pressing treatment, the arrangement of SA layers on the films was largely changed. This was because SA (melting point ~70 °C) was first melted at a high temperature (100 and 150 °C) and was then closely bound to the films under external force pressing. The cross-sectional microstructures of the films also confirmed the morphological changes in SA layers before and after heat pressing treatment. Before heat pressing treatment, the P-S1 and P-S5 films were loosely covered by thick SA layers. After the P-S1 and P-S5 films were heat-pressed, the thick and loosely attached SA layers on the films were compressed into thin and tightly adhered SA layers. Notably, the surfaces of the heat-pressed films were greatly affected by the heat pressing temperature and time. In general, the films heat-pressed at 100 °C had smoother surfaces in comparison with the films heat-pressed at 150 °C. This was because the 100 °C heat pressing treatment made the SA-coated layers distribute uniformly on the films. In contrast, the 150 °C heat pressing treatment disrupted the integrity of SA layers, making some SA layers peel off from the films. Moreover, the destructive effect of heat pressing treatment intensified with the extension of the heat pressing time.

### 3.2. Infrared Spectra of the Films

The compositional changes in the films before and after SA coating and heat pressing treatments were demonstrated by infrared spectroscopy (Figure 2B). The untreated P film showed infrared peaks at 3280, 2924, 1605, and 1148–1018 cm^−1^, individually corresponding to the O–H stretching, C–H stretching, C = C stretching, and C–O–C stretching of PSP constituents (e.g., biopolymers and anthocyanins), sodium alginate, and glycerol [18]. The peak intensity of the P film significantly decreased after 1% of SA coating treatment, because the surface of the P-S1 film was covered by SA layers. At the same time, the P-S1 film displayed the characteristic peaks of SA at 2915 cm^−1^ (methyl stretching), 2847 cm^−1^ (methylene stretching), and 1700 cm^−1^ (C = O stretching) [29,30]. Cai et al. [27] also reported that the infrared spectrum of starch nanofibrous film was changed by SA coating. Notably, the characteristic peaks of the P film disappeared after 5% SA coating treatment, indicating that the surface of P-S5 film was totally covered by thick SA layers. As a result, only the characteristic peaks of SA were observed in the P-S5 film. After the P-S1 and P-S5 films were subjected to heat pressing treatment, the characteristic peaks of the original P film partially recovered. This was because the SA-coated layers became thin after heat pressing treatment. It was worth noting that the heat pressing temperature and time had significant impacts on the infrared spectra of the heat pressing-treated films. As for the films heat-pressed at 100 °C, their infrared spectra were barely affected by the heat pressing time. As for the films heat-pressed at 150 °C, they showed decreased SA peaks with the extension of the heat pressing time. This was because the 150 °C heat pressing treatment seriously disrupted the integrity of SA layers, making some SA layers peel off from the films.

### 3.3. Crystalline Characteristics of the Films

The crystalline characteristics of the films before and after SA coating and heat pressing treatments are presented in Figure 2C. The untreated P film exhibited some weak crystalline peaks at 17.3°, 20.2°, and 22.1°, which were attributed to cellulose and semi-cellulose in PSP [18]. The crystalline peaks of P film were retained after SA coating treatment. Meanwhile, the resultant P-S1 and P-S5 films showed the crystalline peaks of SA at 6.7° and 11.2° [29,31]. The P-S5 film had a higher crystalline degree than the P-S1 film, because the P-S5 film was covered by thicker SA layers. A similar phenomenon was reported by Yun et al. [14] in SA-coated passion fruit peel powder-based films. The crystalline characteristics of P-S1 and P-S5 films were greatly changed after heat pressing treatment. The films heat-pressed at 100 °C displayed decreased crystalline peaks of SA, because the SA-coated layers became thin after heat pressing treatment. However, the crystalline peaks of SA almost disappeared in the films heat-pressed at 150 °C, which was due to the destructive effect of high temperature treatment (150 °C) on the SA-coated layers. After the P-S5 film was heat-pressed at 100 °C and 150 °C, the resultant films showed decreased crystalline peaks of SA with the extension of the heat pressing time. The above results reveal that the crystalline characteristics of the films were influenced by the SA coating concentration, heat pressing temperature, and heat pressing time, which agrees with the results for microstructures (Figure 2A) and infrared spectra (Figure 2B).

### 3.4. Color and Transparency of the Films

**Figure 3:**
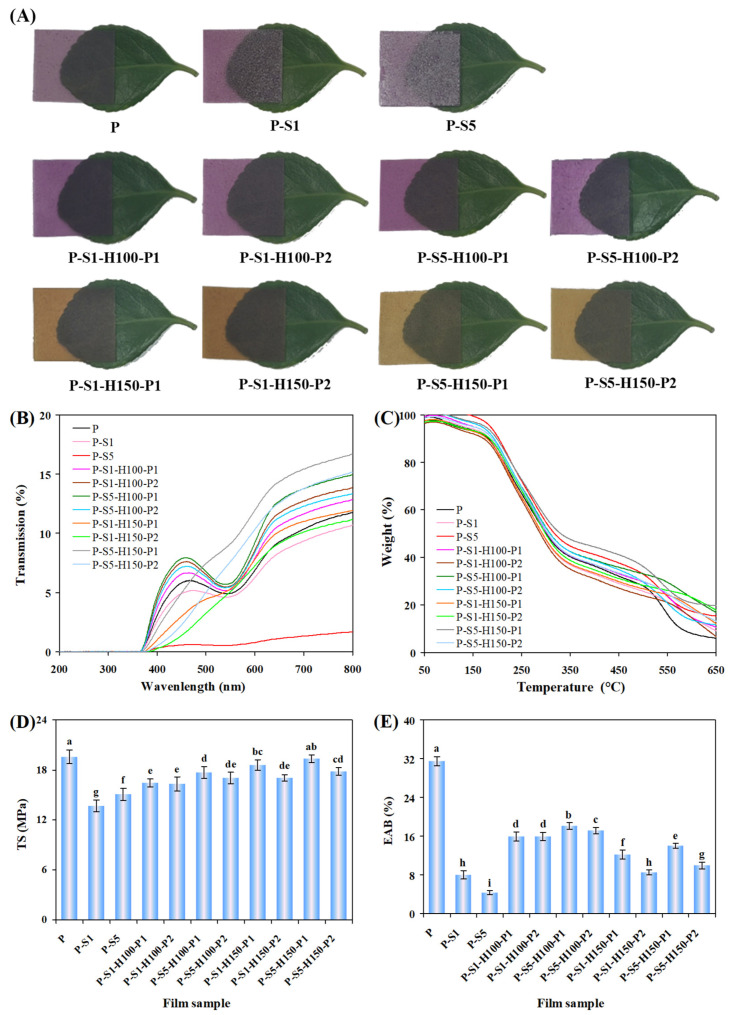
The color (**A**), UV–Vis light transmittance (**B**), thermogravimetric curves (**C**), TS (**D**), and EAB (**E**) of the films. Each value represents mean ± SD (*n* = 6 for TS and EAB). Different lowercase letters signify statistically significant differences (*p* < 0.05).

As shown in Figure 3A and Table 1, the untreated P film was purple with an L* value of 60.47, an a* value of 18.01, and a b* value of −0.65. The purple color of the P film was retained after SA coating treatment. However, the SA-coated layers decreased the transparency of theP-S1 and P-S5 films. Yu et al. [21] also found that chitosan films coated with SA had reduced transparency. Since the P-S5 film was covered by thicker SA layers, the P-S5 film showed a lower transparency than the P-S1 film. After the P-S1 and P-S5 films were heat-pressed at 100 °C, the purple color of the films deepened and the films showed elevated a* values (28.27–32.05). This was probably because the 100 °C heat pressing treatment induced the partial degradation of PSP anthocyanins, producing benzoic acids with a co-pigmentation effect on anthocyanins [32]. The transparency of the P-S1 and P-S5 films was significantly elevated after 100 °C of heat pressing treatment, because the thick SA layers were compressed into thin layers. Meanwhile, the characteristic absorption band of PSP anthocyanins (~550 nm) was largely retained in the films heat-pressed at 100 °C (Figure 3B). In addition, the color and transparency of the films heat-pressed at 100 °C were little impacted by the heat pressing time. Notably, the films heat-pressed at 150 °C were brown and showed high b* values (36.91–51.37). Meanwhile, the characteristic absorption band of PSP anthocyanins (~550 nm) almost disappeared in the films heat-pressed at 150 °C. The results indicate that PSP anthocyanins in the films were seriously destroyed by the 150 °C heat pressing treatment. The brown color of the films heat-pressed at 150 °C was attributed to the thermal degradation products of PSP anthocyanins, especially chalcone [32]. Moreover, the brown color of the films deepened with the extension of the heat pressing time, which was demonstrated by the increased b* values of the films (44.48 for P-S1-P150-H1 film → 51.37 for P-S1-P150-H2 film, and 36.91 for P-S5-P150-H1 film → 49.47 for P-S5-P150-H2 film).

### 3.5. Thermal Property of the Films

The thermal property of the films before and after SA coating and heat pressing treatments is displayed in Figure 3C and Table 2. The thermal decomposition processes of the films were composed of three stages: (I) the evaporation of moisture within the films [18], leading to 2.47–7.60% weight losses; (II) the degradation of glycerol, biopolymers, anthocyanins and SA [18,29,30], leading to 52.78–61.03% weight losses; and (III) the carbonization of remaining materials in the films [33], resulting in 17.24–31.06% weight losses. The predominant decomposition of the films was located at the second stage, with the maximum decomposition rates of the films appearing at 219–274 °C (Figure S1). The untreated P film displayed a single decomposition rate peak at 231 °C. After SA coating treatment, the films clearly showed a new decomposition rate peak at 285 °C, corresponding to the decomposition of the loosely attached SA layers [30]. At the same time, two decomposition rate peaks obviously separated in the P-S1 and P-S5 films. However, after heat pressing treatment, the separation of these two decomposition rate peaks became inapparent. This was because heat pressing treatment made the SA-coated layers closely bind with the films. Notably, the decomposition of the films was associated with the SA coating concentration. In general, the films coated with 5% of SA showed lower decomposition than the films coated with 1% of SA, indicating thicker SA layers had a stronger protective effect on the films. A similar result was reported by Bu et al. [20] in SA-coated gelatin-based nanocomposite films. At 650 °C, the untreated P film showed a lower weight residue than the other films, further confirming that the SA-coated layers slowed down the decomposition of the films.

### 3.6. Mechanical Property of the Films

TS and EAB provide information about the anti-deformation ability and ductility of the films, respectively. As shown in Figure 3D, the TS of the untreated P film was 19.56 MPa, which approached the TS of the films prepared from the steamed PSP [18]. After SA coating treatment, the TS of the films decreased to 13.68−15.07 MPa. This was caused by the dehydration of the films during SA coating, where the interactions between film components were greatly reduced. A similar trend was observed in bagasse cellulose-based papersheet coated with SA [34]. Oppositely, the TS of the films increased to 16.30–19.36 MPa after the films were further subjected to heat pressing treatment. This was because heat pressing treatment reduced the free volume in the films and elevated the molecular interactions between film components. He et al. [15] also demonstrated that SA coating and heat pressing treatments elevated the TS of the regenerated cellulose films. Notably, the TS of the heat-pressed films increased with the elevation of the heat pressing temperature, because the internal compactness of the films was increased under 150 °C heat pressing treatment. However, the TS of the films heat-pressed at 150 °C showed a downward trend when the heat pressing time was extended from 1 min to 2 min, which was because SA layers peeled off from the films with the extension of the heat pressing time. Notably, the TS of PSP/PET composite films was better than that of passion fruit peel powder films coated with SA [14], PSP powder/SA films [17], and starch/gelatin/PSP anthocyanin films [19].

As presented in Figure 3E, the untreated P film displayed 31.49% EAB, indicating that the P film had good ductility. After SA coating treatment, the EAB of the films remarkably decreased to 4.35–8.03%, which was attributed to the dehydration effect of ethanol in the SA solution. Other researchers also reported that the EAB of passion fruit peel powder-based film and bagasse cellulose-based paper decreased after SA coating [14,34]. The EAB of P-S1 and P-S5 films significantly increased after heat pressing treatment, indicating that heat pressing treatment improved the ductility of SA-coated films to some extent. SA, as a plasticizer, could partially enter the films during heat pressing treatment, thereby promoting the fluidity of biopolymer chains [20,35]. When the SA coating concentration increased from 1% to 5%, the amount of SA entering the films increased. Meanwhile, the films coated with 5% of SA had thicker SA layers, which could protect the evaporation of moisture (a good plasticizer) from the films. Therefore, under the same heat pressing treatment conditions, the films coated with 5% of SA showed a higher EAB than the films coated with 1% of SA. The EAB of the films was also affected by the heat pressing temperature, with the films heat-pressed at 150 °C showing a lower EAB than the films heat-pressed at 100 °C. This was because heat pressing treatment at 150 °C resulted in more serious moisture evaporation and SA layer destruction. The films heat-pressed at 150 °C showed a decreased EAB with the extension of the heat pressing time, further confirming the adverse impact of 150 °C heat pressing treatment on the ductility of the films.

### 3.7. MC of the Films

**Figure 4:**
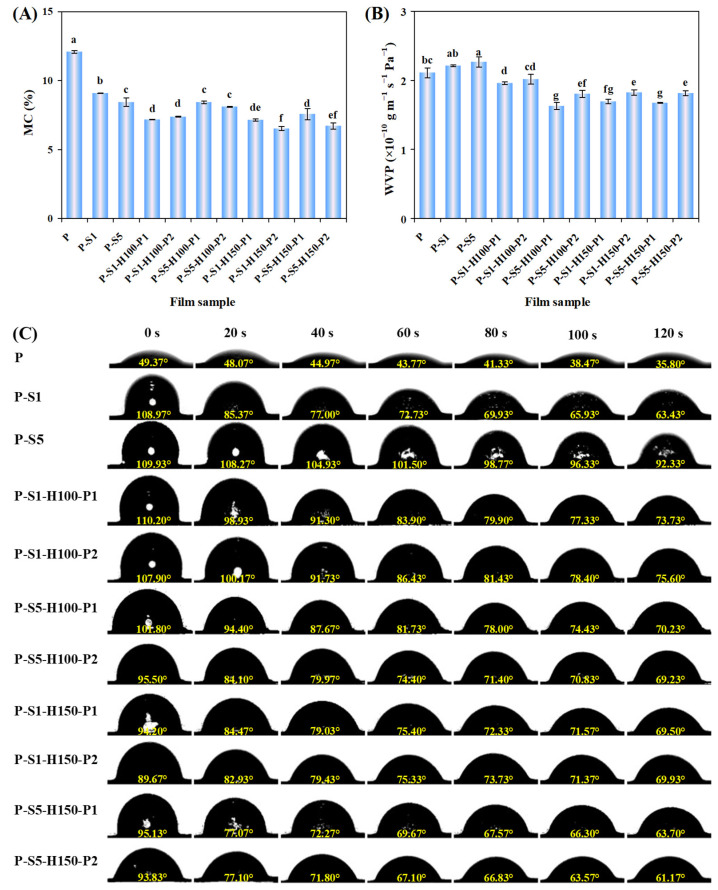
The MC (**A**), WVP (**B**), and dynamic WCA (**C**) of the films. Each value represents the mean ± SD (*n* = 3 for MC and WVP, and *n* = 6 for WCA). Different lowercase letters signify statistically significant differences (*p* < 0.05).

The MC of the films before and after SA coating and heat pressing treatments is presented in Figure 4A. The untreated P film displayed 12.10% MC. However, the MC of the films decreased to 9.10% and 8.44% after coating with 1% and 5% SA, respectively. This was because ethanol in the SA coating solutions had a dehydration effect on the films [14]. The MC of the P-S1 film slightly decreased after the film was heat-pressed at 100 °C, which was caused by the evaporation of moisture from the film. However, the MC of the P-S5 film was almost unchanged at 100 °C heat pressing treatment, which was attributed to the thick SA layers being able to prevent moisture evaporation. As for the films heat-pressed at 100 °C, their MC was not significantly affected by the heat pressing time. Compared with the films heat-pressed at 100 °C, the films heat-pressed at 150 °C showed a lower MC. Meanwhile, the MC of the films heat-pressed at 150 °C continuously decreased with the extension of the heat pressing time. This was because the integrity of SA layers was destroyed under 150 °C heat pressing treatment. At the same time, high temperature conditions (150 °C) accelerated moisture evaporation from the films.

### 3.8. WVP of the Films

WVP is a critical indicator for films in regulating moisture movement between the surrounding environment and food. Films with a low WVP can control the exchange of moisture inside and outside the packaging, thus reflecting the moisture resistance of films [36]. As shown in Figure 4B, the WVP of the untreated P film was 2.109 × 10^−10^ g m^−1^ s^−1^ Pa^−1^. After the P film was coated with 1% and 5% of SA, the WVP of the films slightly increased to 2.21 × 10^−10^ g m^−1^ s^−1^ Pa^−1^ and 2.27 × 10^−10^ g m^−1^ s^−1^ Pa^−1^, respectively. This was caused by the dehydration of the films in SA coating solutions containing ethanol, producing films with loose internal structures for moisture transmission. In addition, the SA layers loosely covered on the P-S1 and P-S5 films provided several channels to entrap moisture. After the P-S1 and P-S5 films were subjected to heat pressing treatment, the WVP of the films decreased by 4.44–22.78%. This was because the internal structures of the films became compact under heat pressing. Meanwhile, SA layers were tightly adhered in the heat-pressed films, which greatly blocked moisture transmission through the films. In addition, the interior of the films was partially filled with SA after heat pressing treatment, which reduced the path of moisture transmission [34]. Notably, the WVP of the films was affected by the SA concentration, heat pressing temperature, and heat pressing time. Since the P-S5 film was covered by thicker SA layers than the P-S1 film, the P-S5 film exhibited a lower WVP after the film was heat-pressed at 100 °C. Wei et al. [37] also observed that the WVP of SA-coated deacetylated konjac glucomannan film decreased with increasing SA concentration. However, after the P-S1 and P-S5 films were heat-pressed at 150 °C, the produced films did not show obvious WVP differences. This was because the integrity of the SA-coated layers was seriously disrupted by 150 °C heat pressing treatment. This was confirmed by the elevated WVP in the films heat-pressed at 150 °C with the extension of the heat pressing time.

### 3.9. WCA of the Films

The WCA is a key parameter reflecting the surface wettability of the films, which is closely related to the roughness and surface energy of the films [10]. The dynamic WCA of the films before and after SA coating and heat pressing treatments is displayed in Figure 4C. The untreated P film showed an initial WCA of 49.37° and a final WCA of 35.80° at 120 s, revealing that the P film was highly hydrophilic. This was because the P film contained several hydrophilic substances, such as starch, sodium alginate, glycerol, and anthocyanins [18]. After the P film was coated with 1% and 5% SA, the WCA of the film increased to 108.97° and 109.93°, respectively. Several previous studies also documented the elevation of WCA in polyvinyl alcohol film [28], chitosan film [21], and deacetylated konjac glucomannan film [37] coated with SA. The reduced surface wettability of the films was attributed to the SA-coated layers having low surface energy [27]. Meanwhile, the SA-coated layers created rough surfaces for the films, providing several voids at the interface of the water drops and the films [20]. The space between SA layers could trap air to form air cushion regions, further reducing the surface wettability of the films [15]. With the extension of the contact time, the WCA of the P-S5 film declined more slowly than that of the P-S1 film, which was because the P-S5 film was covered with thicker SA layers. After heat pressing treatment at 100 °C, the films coated with 1% SA showed a higher WCA than the films coated with 5% SA. This indicated that excessively thick SA layers were not essential in producing heat-pressed films with a low surface wettability. As for the films heat-pressed at 100 °C, their WCA was higher than 65° at 120 s, demonstrating that these films had good hydrophobicity. As reported, heat pressing treatment made the SA-coated layers melt and re-crystallize into micro-/nano-structures [15]. The re-crystallized SA was evenly distributed on the films, thus endowing the films with good hydrophobicity [38]. Notably, the films heat-pressed at 150 °C had a lower initial WCA than the films heat-pressed at 100 °C, which was because the integrity of the SA-coated layers was seriously destroyed under 150 °C heat pressing treatment.

### 3.10. Stability of the Films in Water

**Figure 5:**
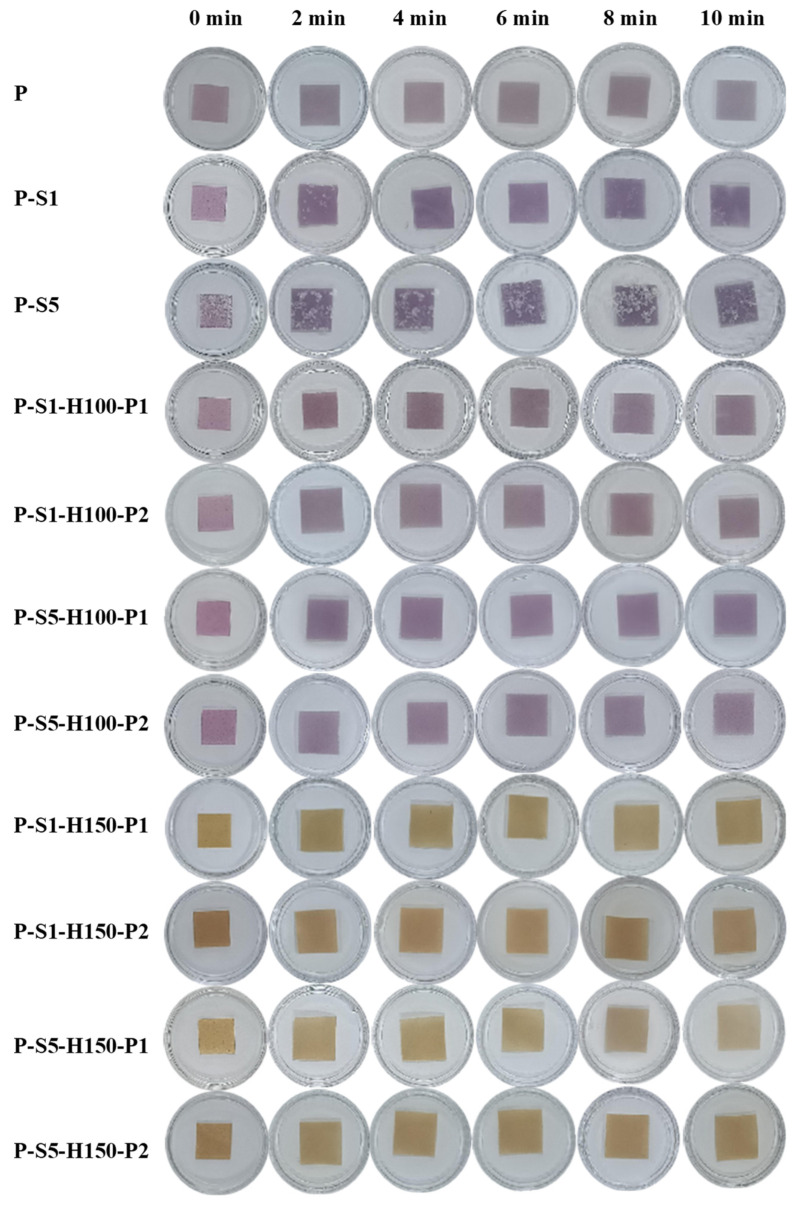
The swelling behavior of the films in distilled water.

**Figure 6:**
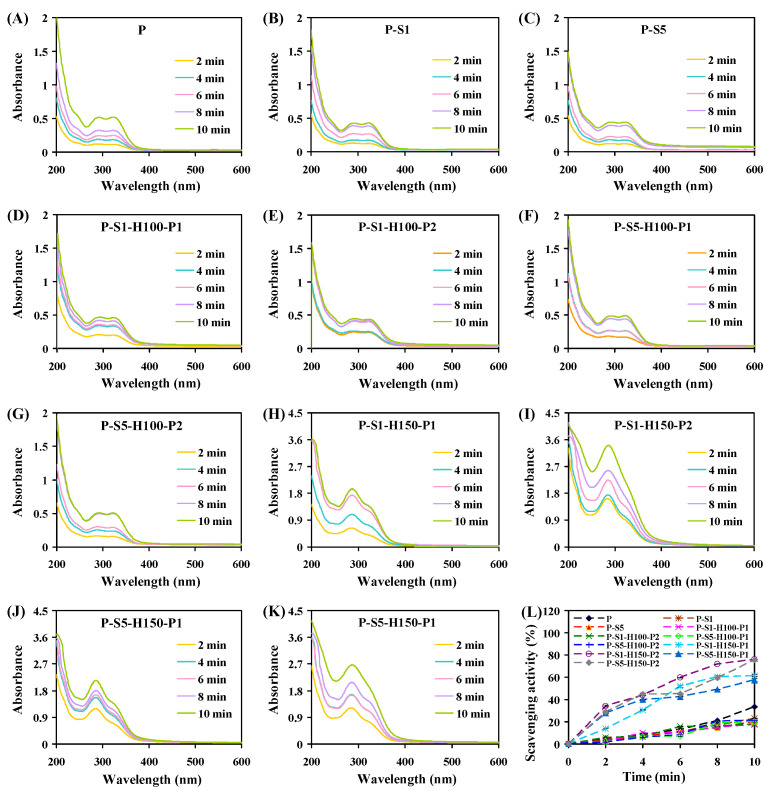
The releasing behavior of the films in distilled water as detected by UV–Vis spectroscopy (**A**–**K**) and the DPPH scavenging test (**L**).

The swelling behavior of the films in water is presented in Figure 5. All films quickly swelled in 2 min. After that, the swelling of the films slowed down. At 10 min, all the films still maintained their integrity. When the P-S1 and P-S5 films were soaked in water, the loosely attached SA layers were gradually separated from the films. After the films were further subjected to heat pressing treatment, the SA-coated layers were compressed and closely bound to the films. As a result, the compressed SA layers were not separated from the heat-pressed films. The releasing behavior of the films was monitored by scanning the UV–Vis absorption spectra of film leachates (Figure 6A–K). The UV–Vis spectrum of the P film leachate displayed two shouldered peaks at 289 and 322 nm, corresponding to anthocyanins. Meanwhile, the peak intensity of the P film leachate increased with the extension of the soaking time, indicating that anthocyanins were continuously released into water. As compared to the P film, the P-S1 and P-S5 films showed relatively lower releasing rates. This revealed that SA coating treatment delayed the release of anthocyanins to some extent, which was also reported in cellulose film coated with SA [15]. The 100 °C heat pressing treatment had little impact on the releasing behavior of the films. However, the releasing behavior of the films was remarkably changed under 150 °C heat pressing treatment. On one hand, the UV–Vis spectra of film leachates changed and displayed a predominant peak at 285 nm. On the other hand, the films heat-pressed at 150 °C showed more leachates than the films heat-pressed at 100 °C. In addition, the releasing behavior of the films heat-pressed at 150 °C accelerated with the extension of the heat pressing time. This was because the 150 °C heat pressing treatment induced the degradation of anthocyanins [32]. Meanwhile, the integrity of SA layers was seriously destroyed under 150 °C heat pressing treatment, meaning that the degraded products of anthocyanins were easily released from the films.

The releasing behavior of the films was also determined by detecting the DPPH scavenging activity of film leachates (Figure 6L). In general, the P film leachate showed similar DPPH scavenging activity with the P-S1, P-S5, P-S1-H100-P1, P-S1-H100-P2, P-S5-H100-P1, and P-S5-H100-P2 film leachates. This indicated that the releasing behavior of the films was not greatly impacted by SA coating and 100 °C heat pressing treatments. In contrast, the leachates from the films heat-pressed at 150 °C displayed a higher DPPH scavenging activity, which was related to the release of anthocyanin-degraded products from the films. This was consistent with the results of UV–Vis spectral detection (Figure 6H–K), showing that the releasing behavior of the films accelerated under 150 °C heat pressing treatment.

### 3.11. Biodegradability of the Films

**Figure 7:**
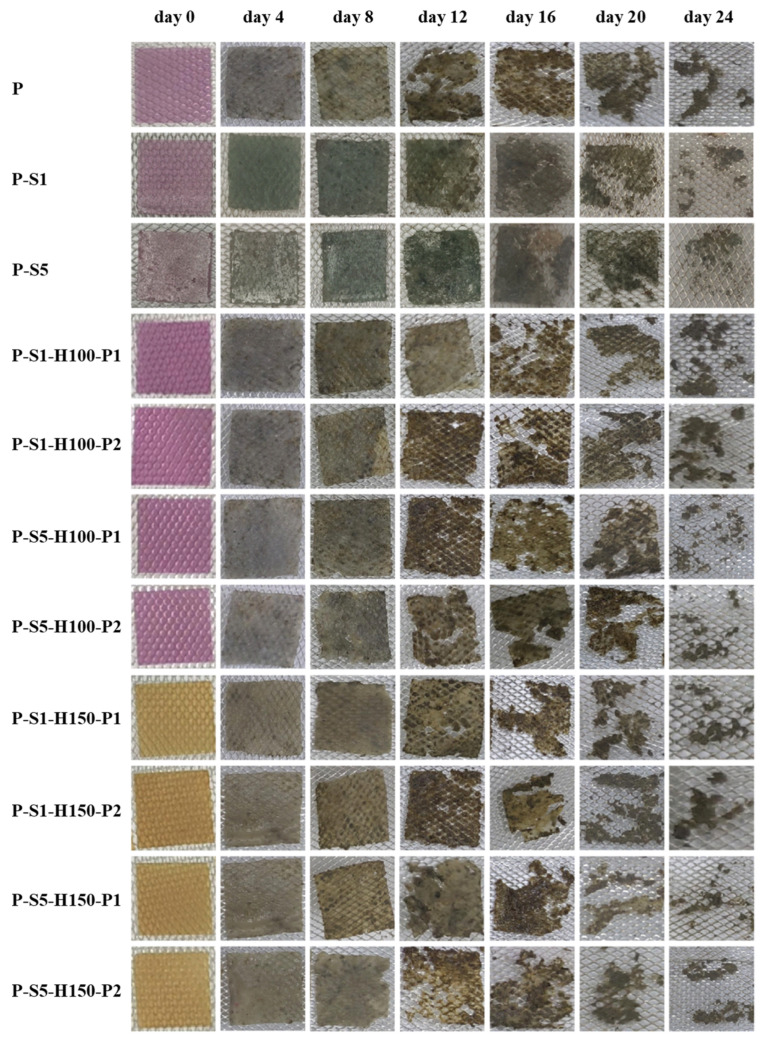
The biodegradation of the films in natural soil over 24 days.

The biodegradation processes of the films were reflected by the morphological changes in the films in natural soil (Figure 7). The original purple-colored films faded within four days due to the loss of anthocyanins in moist soil. Most films were intact within eight days and started to biodegrade thereafter. Some differences were noted in the morphological changes in the films, indicating that SA coating and heat pressing treatments had influenced the biodegradation processes of the films. Notably, the P-S1 and P-S5 films biodegraded more slowly than the P film, which was because the thick SA layers on the P-S1 and P-S5 films formed barriers between soil microorganisms and the films. Yun et al. [14] also reported that the biodegradation of passion fruit peel powder-based films was delayed after SA coating treatment. Compared to the P-S1 and P-S5 films, the heat-pressed films showed quicker biodegradation. This was because the SA-coated layers became thin after heat pressing treatment, making the films more easily accessed by soil microorganisms. Since the SA-coated layers were disrupted by the 150 °C heat pressing treatment, the films heat-pressed at 150 °C biodegraded more rapidly than the films heat-pressed at 100 °C. Although the biodegradation processes of the films were retarded after SA coating and heat pressing treatments, all the films displayed good biodegradation performances on the 24th day. He et al. [15] also demonstrated that SA-coated cellulose films could be biodegraded by more than half within 52 days. This indicated the films were still biodegradable and environmentally friendly after SA coating and heat pressing treatments.

### 3.12. Color Changeability of the Films

**Figure 8:**
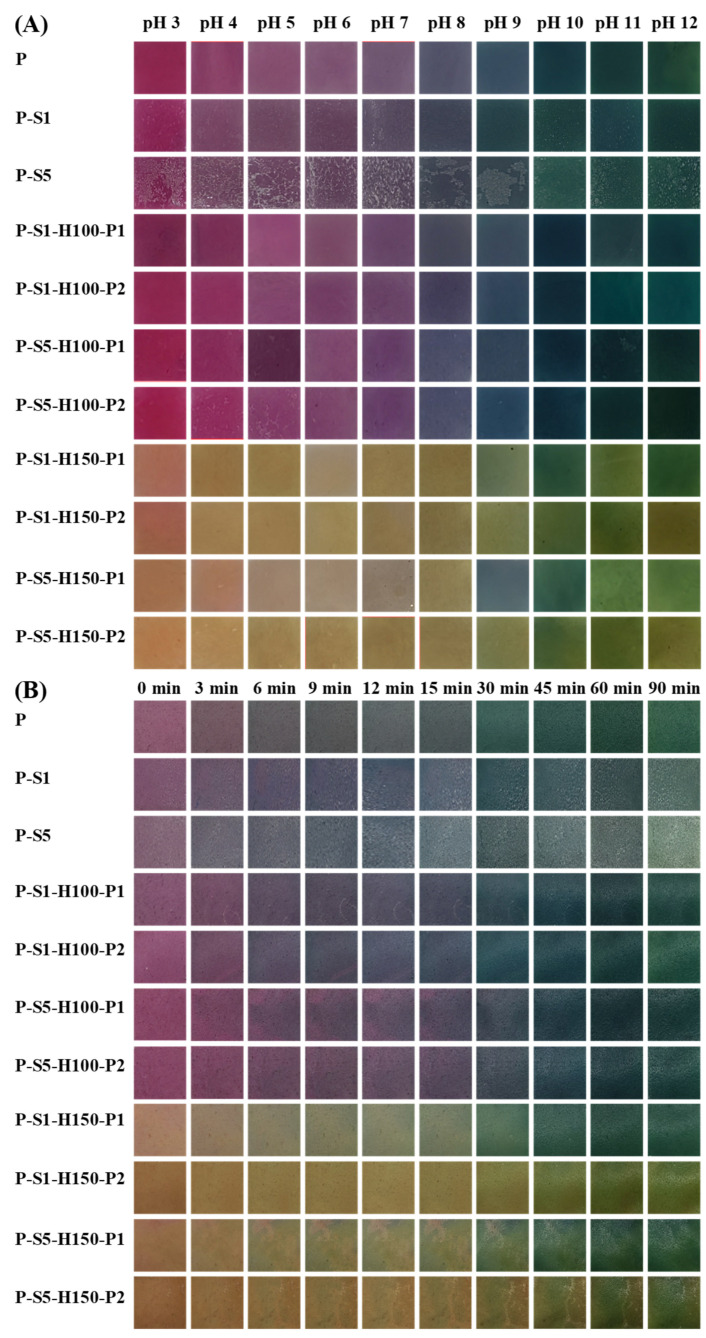
The color changeability of the films in pH 3–12 buffers (**A**) and 0.1 mol/L of ammonia vapor (**B**).

The color changeability of the films is a key factor reflecting the potential of the films in intelligent packaging [39]. The color changeability of the films was tested in pH 9–12 buffers and 0.1 mol/L ammonia vapor. The untreated P film exhibited a color transition trend from red to purple, blue-violet, blue, and green when the pH increased from 3 to 12 (Figure 8A). Meanwhile, the purple P film first turned blue and then turned green in ammonia vapor (Figure 8B). Similar color-changing trends were observed in PSP powder/SA films [18]. The color changeability of the P film was attributed to anthocyanins in PSP [18]. Since the P-S1 and P-S5 films were unevenly covered by thick SA layers, the observation of films’ color changes in buffers and ammonia vapor was negatively affected. After the P-S1 and P-S5 films were heat-pressed at 100 °C, the color changeability of the films was promoted. This was because heat pressing treatment reduced the thickness of SA layers, making them closely bind to the films and evenly distribute on the films. Notably, the films heat-pressed at 100 °C showed superior color changeability in comparison with the P film, which was attributed to the co-pigmentation effect formed between anthocyanins and their thermal degradation products [32]. However, after the P-S1 and P-S5 films were heat-pressed at 150 °C, anthocyanins in the films were seriously destroyed. As a result, the films heat-pressed at 150 °C almost lost color changeability, with only the P-S1-H150-P1, and P-S5-H150-P1 films showing color variation in pH 9–12 buffers and ammonia vapor at 30–90 min.

### 3.13. The Mechanism of Action of SA Coating and Heat Pressing Treatments

**Figure 9:**
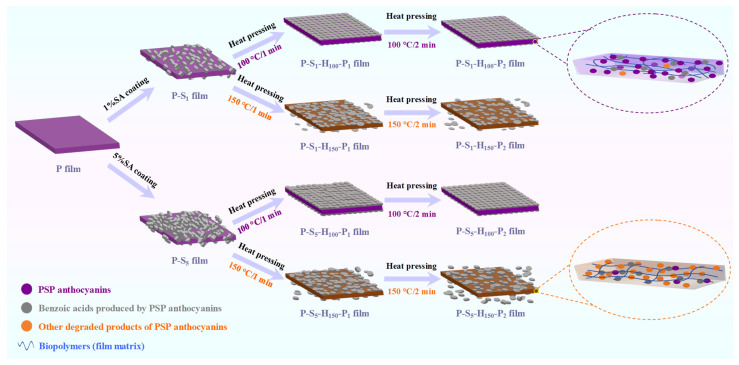
The mechanism of action of SA coating and heat pressing treatments.

Based on the structural characteristics, physical properties, and color changeability of the films stated above, the mechanism of action of SA coating and heat pressing treatments is summarized and illustrated in Figure 9. After the P film was soaked in 1% and 5% SA coating solutions, the surfaces of the obtained P-S1 and P-S5 films were loosely covered by massive SA layers. The SA-coated layers created rougher surfaces and reduced the surface energy of the films, meaning that the P-S1 and P-S5 films showed reduced surface wettability. However, the thick SA layers on the P-S1 and P-S5 films negatively affected the transparency, biodegradability, and color changeability of the films. Moreover, the P-S1 and P-S5 films were dehydrated by ethanol in SA coating solutions, which decreased the MC, water vapor blocking ability, and mechanical property of the films. After the P-S1 and P-S5 films were heat-pressed at 100 °C, the SA-coated layers were compressed and uniformly distributed on the films. As a result, the films heat-pressed at 100 °C presented promoted transparency and biodegradability. At the same time, heat pressing treatment at 100 °C reduced the free volume in the films and elevated the molecular interactions between film components, producing films with an enhanced mechanical property and water vapor blocking ability. Notably, the 100 °C heat pressing treatment induced the partial degradation of PSP anthocyanins, producing benzoic acids with a co-pigmentation effect on anthocyanins. Therefore, the films heat-pressed at 100 °C displayed a vivid purple color and elevated color changeability. After the P-S1 and P-S5 films were heat-pressed at 150 °C, the integrity of SA layers was disrupted and some SA layers peeled off from the films. As a result, the films heat-pressed at 150 °C showed a lower EAB, MC, and WCA but a higher leaching potential than the films heat-pressed at 100 °C. Meanwhile, anthocyanins in the films were seriously destroyed, meaning that the films presented a brown color and lost color changeability.

### 3.14. Shrimp Freshness Monitoring Ability of the Films

**Figure 10:**
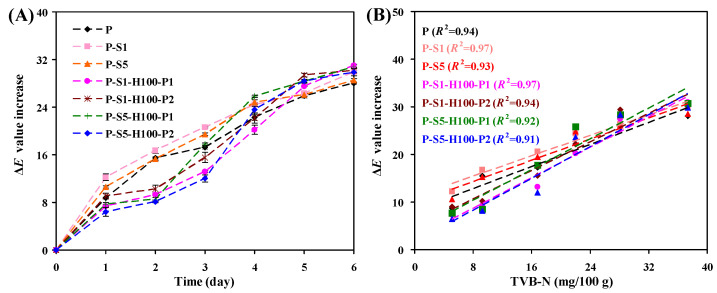
The Δ*E* value increase of the films during shrimp storage at 4 °C for 6 days (**A**) and correlation analysis between the Δ*E* value increase of the films and the TVB-N level of shrimps (**B**).

The intelligent packaging potential of the films was validated by applying the films for the monitoring of shrimp freshness, where the color changes in the films were recorded during the cold storage of shrimps. Meanwhile, the freshness of shrimps was tracked by detecting the TVB-N of shrimps. As indicated in Table 3, shrimps were no longer suitable for consumption on the fourth day, because the TVB-N of shrimps had exceeded 20 mg/100 g. On the fourth day, all the films except for those heat-pressed at 150 °C displayed noticeable color changes, i.e., from purple to blue-violet. The color changes in the films were reflected by the Δ*E* value of the films, with the Δ*E* value of the films maintaining an increasing trend (Figure 10A). Correlation analysis demonstrated that the Δ*E* value increase in the films was positively correlated with the TVB-N of shrimps, with correlation coefficients ranging from 0.91 to 0.97 (Figure 10B). Notably, the films heat-pressed at 100 °C presented more vivid color changes, which agreed with the better color changeability of these films (Figure 8). This demonstrated that the intelligent packaging potential of the films was promoted after heat pressing treatment at 100 °C. Since anthocyanins were largely decomposed at 150 °C, the films heat-pressed at 150 °C exhibited a brown color all the time and lost their shrimp freshness monitoring ability.

## 4. Conclusions

In this study, surface-hydrophobized PSP films were prepared by SA coating combined with heat pressing treatments. SA coating treatment greatly enhanced the surface hydrophobicity of the films but negatively affected the transparency, mechanical property, and color changeability of the films. Further heat pressing treatment improved the transparency and mechanical property of the SA-coated films without causing a loss in the surface hydrophobicity of the films. The heat pressing temperature greatly affected the structural characteristics, physical properties, and color changeability of the films. The SA-coated layers were compressed and tightly adhered to the films under heat pressing treatment at 100 °C. The films heat-pressed at 100 °C even showed better color changeability and shrimp freshness monitoring ability than the untreated PSP films. However, the integrity of the SA-coated layers was seriously disrupted by heat pressing treatment at 150 °C. The films heat-pressed at 150 °C lost their color changeability and shrimp freshness monitoring ability and even showed a higher leaching potential than the untreated PSP films. The biodegradability of the films was not greatly affected by SA coating and heat pressing treatments. This study reveals that the films heat-pressed at 100 °C have good intelligent packaging potential.

## Figures and Tables

**Table 1:** Color parameters, including the L*, a*, b*, and Δ*E* of the films.

Films	L*	a*	b*	Δ*E*
P	60.47 ± 0.31 ^b^	18.01 ± 0.45 ^e^	−0.65 ± 0.03 ^e^	37.62 ± 0.45 ^i^
P-S1	56.06 ± 0.12 ^d^	19.71 ± 0.15 ^d^	−3.20 ± 0.14 ^f^	42.15 ± 0.17 ^h^
P-S5	53.37 ± 0.12 ^g^	19.84 ± 0.25 ^d^	−2.93 ± 0.01 ^f^	63.36 ± 0.14 ^c^
P-S1-H100-P1	52.41 ± 0.32 ^h^	32.05 ± 0.31 ^a^	−9.72 ± 0.15 ^j^	52.22 ± 0.42 ^e^
P-S1-H100-P2	56.66 ± 0.29 ^c^	28.27 ± 0.15 ^c^	−6.01 ± 0.05 ^g^	46.30 ± 0.24 ^g^
P-S5-H100-P1	54.95 ± 0.03 ^e^	30.00 ± 0.08 ^b^	−8.40 ± 0.07 ^i^	48.85 ± 0.02 ^f^
P-S5-H100-P2	54.10 ± 0.47 ^f^	29.98 ± 0.86 ^b^	−7.15 ± 0.72 ^h^	49.43 ± 0.93 ^f^
P-S1-H150-P1	60.46 ± 0.15 ^b^	12.56 ± 0.03 ^g^	44.48 ± 0.05 ^c^	60.15 ± 0.05 ^d^
P-S1-H150-P2	55.01 ± 0.29 ^e^	16.67 ± 0.41 ^f^	51.37 ± 0.32 ^a^	69.72 ± 0.51 ^a^
P-S5-H150-P1	64.42 ± 0.23 ^a^	11.13 ± 0.78 ^h^	36.91 ± 0.55 ^d^	51.57 ± 0.51 ^e^
P-S5-H150-P2	55.74 ± 0.07 ^d^	16.53 ± 0.17 ^f^	49.47 ± 0.15 ^b^	67.66 ± 0.04 ^b^

Values are given as mean ± SD (*n* = 3). Different letters in the same column indicate significant differences (*p* < 0.05).

**Table 2:** Thermogravimetric data of the films.

Films	Stage I		Stage II		Stage III		*T*_m_ (°C)	Weight Residue at 650 °C (%)
	Temperature Range (°C)	Weight Loss (%)	Temperature Range (°C)	Weight Loss (%)	Temperature Range (°C)	Weight Loss (%)		
P	50–140	5.96	140–391	57.05	391–650	31.06	231	5.93
P-S1	50–151	6.07	151–389	60.32	389–650	24.57	219	9.04
P-S5	50–150	2.47	150–393	55.94	393–650	26.34	222	15.25
P-S1-H100-P1	50–141	4.37	141–392	58.23	392–650	26.81	235	10.59
P-S1-H100-P2	50–143	7.60	143–394	61.03	394–650	24.80	233	6.56
P-S5-H100-P1	50–143	6.54	143–395	54.35	395–650	22.37	274	16.74
P-S5-H100-P2	50–142	3.06	142–396	57.48	396–650	27.86	230	11.61
P-S1-H150-P1	50–142	6.48	142–399	60.09	399–650	21.39	232	12.04
P-S1-H150-P2	50–144	6.30	144–396	58.71	396–650	17.24	270	17.74
P-S5-H150-P1	50–141	2.56	141–395	52.78	395–650	24.94	235	19.72
P-S5-H150-P2	50–142	4.59	142–393	57.98	393–650	24.34	238	13.10

*T*_m_: temperature with the maximum decomposition rate of the films.

**Table 3:** Changes in the TVB-N level of shrimps and the color of films during the cold storage of shrimps.

Time (day)	0	1	2	3	4	5	6
TVB-N level (mg/100 g)	4.11 ± 0.46 ^g^	5.12 ± 0.46 ^f^	9.23 ± 0.50 ^e^	16.78 ± 1.13 ^d^	22.02 ± 0.76 ^c^	28.01 ± 0.47 ^b^	37.33 ± 0.79 ^a^
P	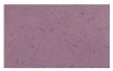	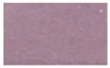	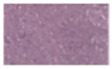	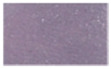	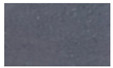	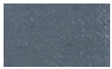	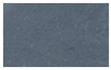
P-S1	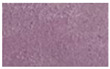	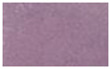	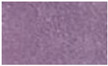	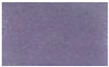	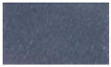	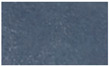	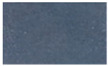
P-S5	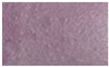	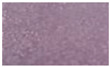	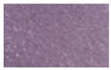	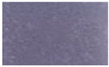	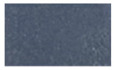	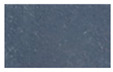	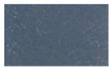
P-S1-H100-P1	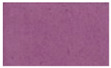	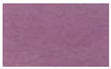	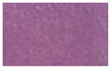	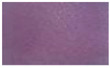	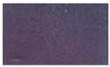	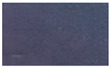	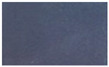
P-S1-H100-P2	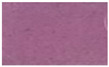	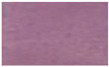	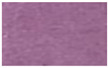	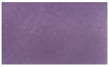	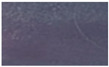	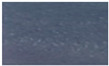	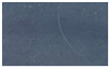
P-S5-H100-P1	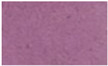	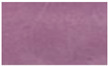	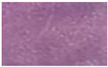	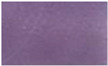	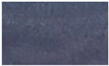	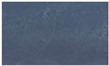	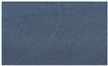
P-S5-H100-P2	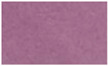	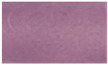	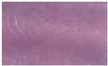	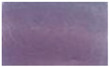	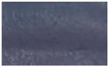	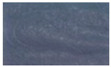	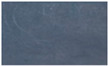
P-S1-H150-P1	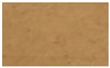	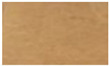	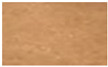	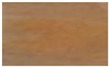	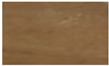	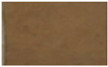	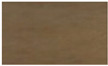
P-S1-H150-P2	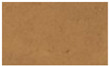	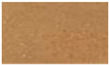	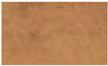	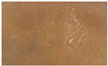	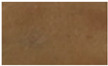	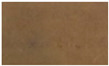	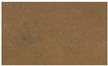
P-S5-H150-P1	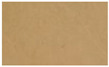	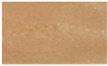	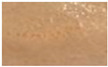	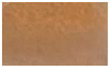	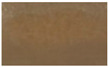	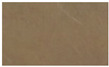	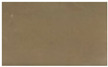
P-S5-H150-P2	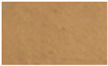	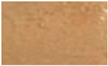	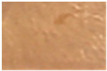	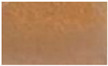	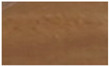	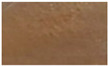	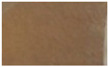

Values are given as mean ± SD (*n* = 3). Different lowercase letters in the same column indicate a significant difference (*p* < 0.05).

## Data Availability

Data will be made available on request from the corresponding author. The data are not publicly available due to privacy restrictions.

## Supplementary Materials

The following supporting information can be downloaded at: https://www.mdpi.com/article/10.3390/foods14071276/s1, Figure S1: The DTG curves of the P (A), P-S1 (B), P-S5 (C), P-S1-H100-P1 (D), P-S1-H100-P2 (E), P-S5-H100-P1 (F), P-S5-H100-P2 (G), P-S1-H150-P1 (H), P-S1-H150-P2 (I), P-S5-H150-P1, (J) and P-S5-H150-P2 (K) films.
