# Supplementary material for: Preparation of Hydrophobic Purple Sweet Potato-Based Intelligent Packaging Films by Stearic Acid Coating and Heat Pressing Treatments
Source: Foods. 2025 Apr 5;14(7):1276. doi: 10.3390/foods14071276 (PMC11988679; doi:10.3390/foods14071276)
Supplement: Supplementary file 1 [file foods-14-01276-s001.zip › foods-3551555-supplementary.pdf]

Supplementary Material:

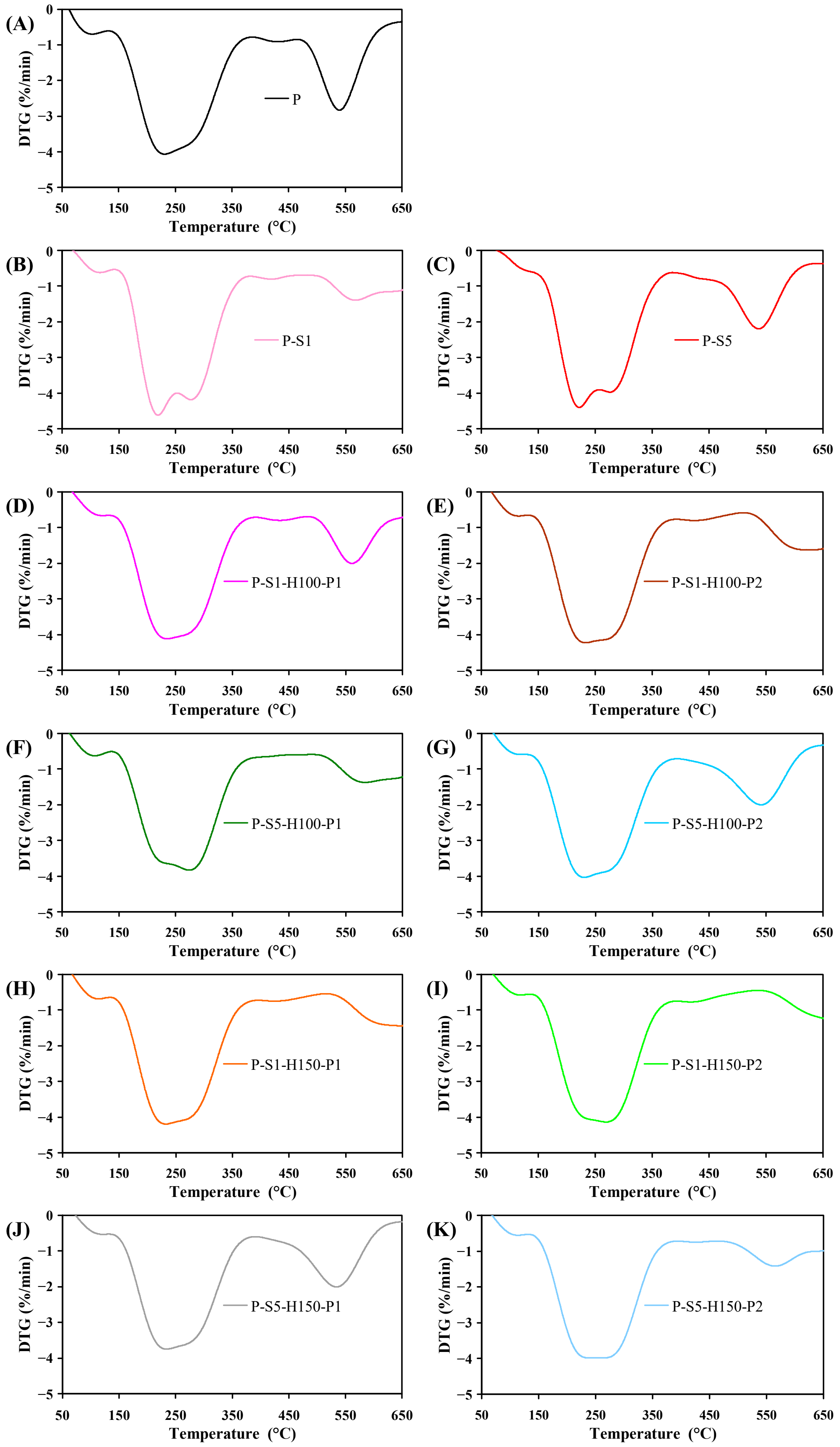

Figure S1. The DTG curves of the P (A), P-S1 (B), P-S5 (C), P-S1-H100-P1(D), P-S1-H100-P2 (E), P-S5-H100-P1(F), P-S5-H100-P2 (G), P-S1-H150-P1 (H), P-S1-H150-P2 (I), P-S5-H150-P1 (J) and P-S5-H150-P2 (K) films.
